# Supplementary material for: Inferring the composition of a mixed culture of natural microbial isolates by deep sequencing
Source: bioRxiv. 2024 Aug 5:2024.08.05.606565. Preprint. [Version 1] doi: 10.1101/2024.08.05.606565 (PMC11326141; doi:10.1101/2024.08.05.606565)
Supplement: Supplement 1 [file NIHPP2024.08.05.606565v1-supplement-1.pdf]

bioRxiv preprint doi: https://doi.org/10.1101/2024.08.05.606565; this version posted August 5, 2024. The copyright holder for this preprint (which was not certified by peer review) is the author/funder, who has granted bioRxiv a license to display the preprint in perpetuity. It is made available under aCC-BY 4.0 International license.

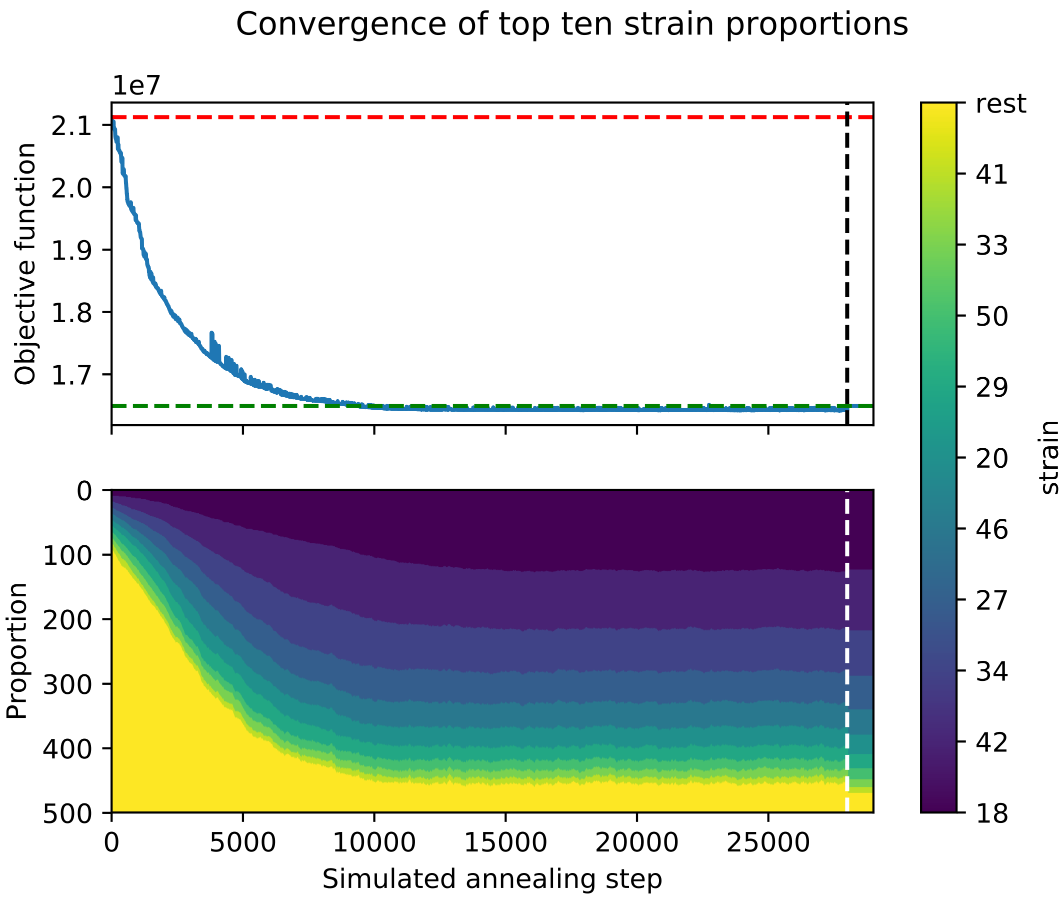

Fig S1. Fitting strain proportions with simulated annealing. (Top) Objective function for each simulated annealing step for a simulated pool (blue line). Values of the objective function are shown as dashed lines for a uniform strain distribution (red) and for the known correct distribution (green). (Bottom) Estimated proportions of the top ten strains or the remaining strains ("rest") plotted as stacked relative proportions for each simulated annealing step. For both the top and bottom plots, the true solution is plotted to the right of the vertial dashed line.

Supporting information

S1 Table. Table of strains used in this work as tab-delimited text file. Publication_ID gives the strain name used in the main text and figures. Additional identifiers that have been associated with a strain are given in Preliminary_ID, Collection_ID, ALT_ID, UCSF_ID, and FASTQ_prefix. Collection details are given in SPECIES, COUNTRY, CITY/LOCATION, STATE/COUNTRY, ISOLATION/DISEASE_INFO, and YEAR. Strains used in the first set of pools, the retesting set of pool, or the GWAS analysis are indicated with "True" in the Pool1, Pool2, or GWAS columns respectively. Previously sequenced strains are indicated by SRA run ID in the Previously_sequenced column. Strains newly sequenced or resequenced in this work are indicated with "True" in the Sequenced column.

bioRxiv preprint doi: https://doi.org/10.1101/2024.08.05.606565; this version posted August 5, 2024. The copyright holder for this preprint (which was not certified by peer review) is the author/funder, who has granted bioRxiv a license to display the preprint in perpetuity. It is made available under aCC-BY 4.0 International license.

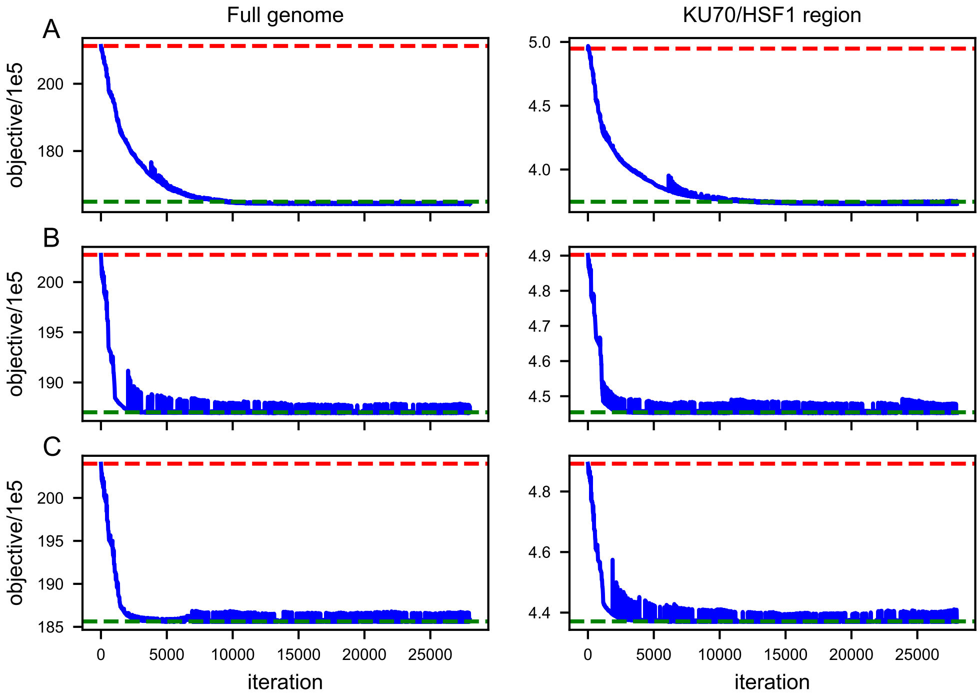

Fig S2. Simulated annealing converges to ground truth for simulated pools. Objective function at each simulated annealing step for the simulated pools A, B, and C (blue). Values of the objective function are shown as dashed lines for a uniform strain distribution (red) and for the known correct distribution (green). Curves are plotted for SNPs identified from the full genome (left) or from the transposon-free region between KU70 and HSF1, representing about 5% of the genome.

S2 File. Code. Zip archive of the python code implementing our fitting method and the Jupyter notebooks required to generate the figures in the paper.

bioRxiv preprint doi: https://doi.org/10.1101/2024.08.05.606565; this version posted August 5, 2024. The copyright holder for this preprint (which was not certified by peer review) is the author/funder, who has granted bioRxiv a license to display the preprint in perpetuity. It is made available under aCC-BY 4.0 International license.

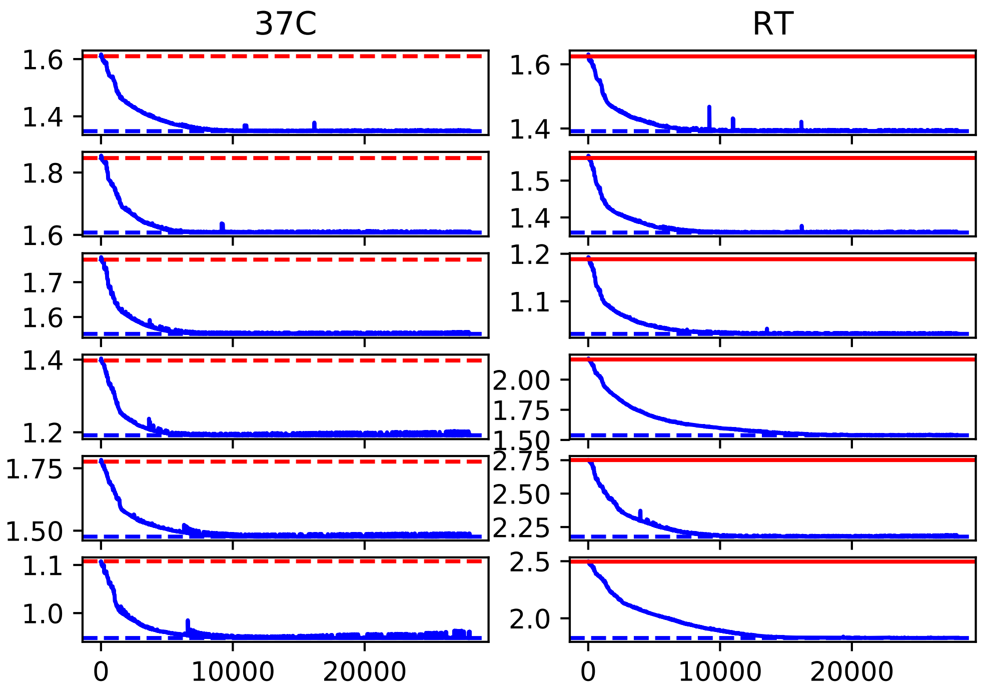

Fig S3. Simulated annealing converges for real pools. For each of 12 pools, the objective function is plotted as a function of the simulated annealing step as a solid blue line with the minimum value as a dashed blue line and the objective function evaluated for a uniform strain distribution plotted as a solid red line.

bioRxiv preprint doi: https://doi.org/10.1101/2024.08.05.606565; this version posted August 5, 2024. The copyright holder for this preprint (which was not certified by peer review) is the author/funder, who has granted bioRxiv a license to display the preprint in perpetuity. It is made available under aCC-BY 4.0 International license.

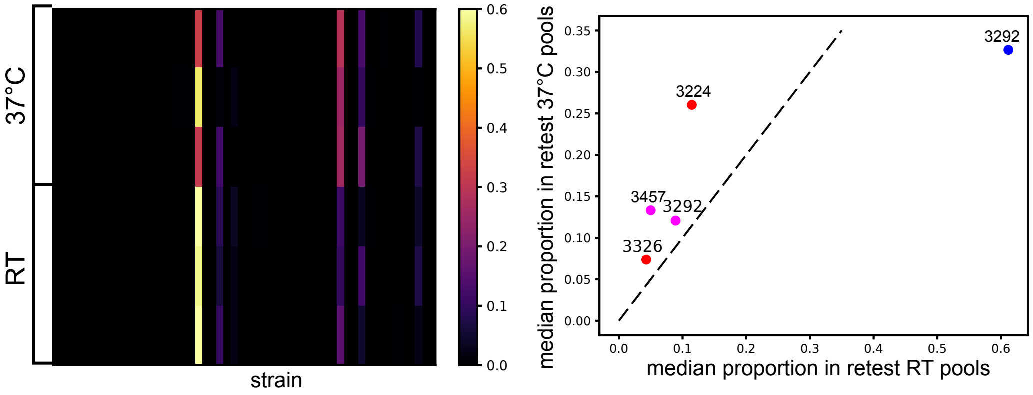

Fig S4. Strain proportions fit from retest pool sequencing. (A) Heatmap showing fit proportions for each of 54 strains (columns) in each of 6 pooled liquid cultures of 5 strains (rows) grown for 14 days at 37°C or RT. (B) Scatter plot of median proportion for each strain in the 37°C vs. RT pools from (A). Strains are colored as in Fig 3B. Dashed line indicates a slope of 1.
